# Heart Rate Variability in Unexplained Syncope Patients Versus Healthy Controls: A Comparative Study

**DOI:** 10.7759/cureus.41370

**Published:** 2023-07-04

**Authors:** Rashmi R Dash, Priyadarsini Samanta, Susnato Das, Anupam Jena, Bandita Panda, Barsha B Parida, Jayanti Mishra

**Affiliations:** 1 Department of Physiology, Kalinga Institute of Medical Sciences, Bhubaneswar, IND; 2 Department of Cardiology, Kalinga Institute of Medical Sciences, Bhubaneswar, IND; 3 Department of Research and Development, Kalinga Institute of Medical Sciences, Bhubaneswar, IND; 4 Department of Physiology, All India Institute of Medical Sciences., Bhubaneswar, IND

**Keywords:** cardiovascular autonomic changes, lf/hf ratio, sympathetic predominance, unexplained syncope, heart rate variability

## Abstract

Background

Syncope or fainting is the sudden and transient loss of consciousness. This could lead to an increase in mortality due to sudden cardiac death or comorbidity in these patients. Heart rate variability (HRV) is a noninvasive bedside procedure for assessing the cardiovascular autonomic function. There may be an abnormal alteration in the HRV parameters in syncope patients. This can be used for looking into cardiovascular autonomic changes in syncope. This would help in early diagnosis and intervention.

Objective

The aim of this present study was to compare the HRV parameters between unexplained syncope patients and age-matched healthy controls and to find a correlation between HRV parameters and cardiovascular parameters like pulse and mean blood pressure.

Materials and methods

A five-minute continuous electrocardiogram (ECG) was recorded and HRV analysis was done by ADInstruments' PowerLab (Oxford, United Kingdom) for 25 cases and 25 controls.

Results

The mean standard deviation of the RR interval (SDRR) in milliseconds was found to be significantly lower in the cases (21.93 ± 3.53) as compared to controls (71.27 ± 27.40). The mean value of the low-frequency to high-frequency ratio (LF/HF) was significantly higher in cases (1.43 ± 0.40) as compared to controls (0.98 ± 1.07). However, there was no significant correlation between the pulse, blood pressure, and HRV measures.

Conclusion

The findings suggest a sympathetic predominance in the cases of unexplained syncope as compared to the controls.

## Introduction

Heart rate variability (HRV) is the physiologic occurrence of fluctuation in the interval between consecutive heartbeats. It is calculated by the variation in the beat-to-beat interval, usually the distance between one R and the consecutive R wave in milliseconds. The sinoatrial node (SA node) receives autonomic and humoral inputs, and hence we observe this variability [1,2]. HRV is a noninvasive procedure for assessing cardiovascular autonomic function. Less heart rate variability signifies disease [3]. Syncope or fainting is the sudden and transient loss of consciousness and postural tone with automatic recovery, which occurs due to temporary insufficiency of cerebral blood flow. Syncope is a common clinical problem that affects up to 3.5% of the general population all over the world. In approximately 40% of cases, the exact cause of syncope remains unknown, and 30% of patients experience recurrent episodes. This leads to an increase in mortality due to sudden cardiac death, comorbidity, and disability-adjusted life years (DALY) [4,5].

Unexplained syncope is defined as a loss of consciousness that cannot be explained by clinical history, physical examination, and a battery of other tests, including neurological examinations. In a few studies, it has been subtly stated that the modulations of the autonomic nervous system on the SA node could play an important role in the pathophysiology of syncope genesis [6,7]. There may be an abnormal alteration in the HRV parameters in syncope patients. This can be used for looking into cardiovascular autonomic changes in syncope and may help as a simple noninvasive bedside tool for early diagnosis and control measures.

Therefore, the primary aim of this study was to compare the HRV parameters between the unexplained syncope patients and the age-matched healthy controls. The secondary objective consisted of finding a correlation between HRV parameters with cardiovascular parameters like pulse and mean blood pressure.

## Materials and methods

Study design

This case-control study was conducted in the Department of Physiology, Kalinga Institute of Medical Sciences (KIMS), Bhubaneswar, Odisha.

Patient recruitment

**Figure 1:**
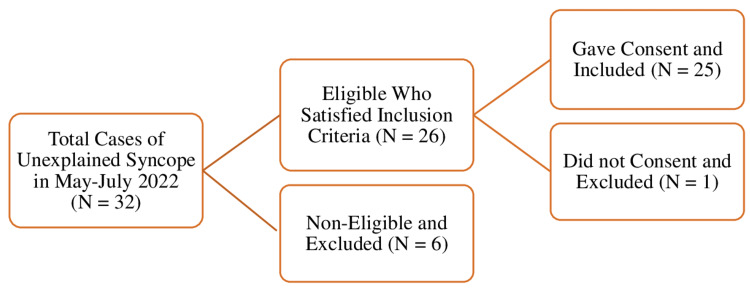
Flow diagram showing the inclusion and exclusion of study participants

After approval by our institutional review board and institutional ethics committee (KIIT/KIMS/IEC/924/2022) and following the declaration of Helsinki, cases of unexplained syncope attending the cardiology outpatient department of our tertiary care hospital were screened and recruited by the cardiologist after thorough history taking and clinical examination. Healthy age-matched controls were recruited from among the office staff and faculties of our institute. Patients with a past history or known causes of syncope or known cardiovascular, respiratory, CNS, neuropsychiatric, or endocrine disorders and a history of chronic medication were excluded from the study. The consecutive number of cases who attended the cardiology outpatient department between May and July 2022 was 32. Twenty-six of them satisfied our inclusion criteria and six were excluded. Twenty-five patients gave written informed consent and were taken as cases (Figure 1). Among the cases, 19 were males and six were females with a mean age of 62.28±5.68. The number of age-matched healthy controls taken was 25 (17 males and 8 females) with a mean age of 60.40±16.92.

Study parameters

Demographic parameters like age and BMI were measured using standard techniques. Pulse was noted manually. Systolic blood pressure and diastolic blood pressure were measured by a mercury sphygmomanometer. HRV was assessed by a Powerlab-26T, Lab Chart 8, HRV version 2.0 by ADInstruments ((Oxford, United Kingdom). The HRV parameter of the time domain taken was the standard deviation of the RR interval (SDRR) and the frequency domain parameter taken was the ratio of low frequency to high frequency (LF/HF ratio).

Methodology

Patients were asked to rest and relax for 10 minutes. They were also told to remove any metallic ornaments or possessions. Then, in the presence of an attendant of the same gender, a five-minute continuous ECG recording was taken using Power Lab 26-T AD instrument. The test was done between 9 to 11 am in order to avoid diurnal variation. The subjects were asked to refrain from tea, coffee, or medications 12 hours prior to the test. One time domain measure is the standard deviation of all normal sinus RR intervals during a five-minute period (SDRR) was noted. One frequency domain parameter, which is a component of HRV, LF/HF ratio was taken into consideration. The LF/HF ratio is calculated by dividing the LF value by the HF value to provide a unit-less ratio. It is a marker of sympathetic nerve activity. HF (0.15-0.4 Hz) power is considered to be mediated mainly by vagal activity while LF (0.04-0.15 Hz) power has been suggested as a predominantly sympathetic modulation [3].

Statistical analysis

All the continuous data were presented in the form of mean ± SD. The normality of the data was tested by the Shapiro-Wilk test. The unpaired t-test between the different parameters and Pearson’s correlation coefficient was determined by using IBM SPSS software version 23.0 (IBM Corp., Armonk, NY). A P-value of less than 0.05 was taken as statistically significant.

## Results

The demographic parameters of 25 syncope cases and 25 healthy subjects are shown in Table 1. Among 25 cases, six were females and 19 were males and in the control, eight were females and 17 were males. The mean age of the control was 60.40±16.92 and that of the cases was 62.28±5.68. The mean BMI of controls and cases was 25.97±0.71 and 26.19±0.64, respectively.

**Table 1:** Demographic parameters between cases and controls NS=non-significant, ⁕=significant (P value≤ 0.05)

	CONTROLS (N=25)	CASES (N=25)	P VALUE
AGE (Years)	60.40 ± 16.92	62.28 ± 5.68	0.100 (NS)
BMI (Body Mass Index)	25.97 ± 0.71	26.19 ± 0.64	0.248 (NS)
GENDER (Male/Female)	17/8	19/6	

The clinical parameters between cases and controls are presented in Table 2. There was no significant difference in systolic blood pressure (SBP) and diastolic blood pressure (DBP) between both groups. The pulse rate was significantly higher in cases as compared to controls. The time domain parameter SDRR was significantly reduced in cases as compared to controls. There was an increase in the values of the LF/HF ratio in the cases, which was statistically significant.

**Table 2:** Clinical parameters between cases and controls NS=non-significant, ⁕=significant (P value≤ 0.05), SBP=systolic blood pressure, DBP=diastolic blood pressure, SDRR=standard deviation of the RR interval, LF/HF=ratio of low frequency to high frequency

	CONTROLS (N=25)	CASES (N=25)	P VALUE
PULSE (bpm)	70.32 ± 7.57	79.56 ± 14.32	0.041^⁕^
SBP (mm Hg)	128.16 ± 6.97	120.4 ± 5.68	0.061 (NS)
DBP (mm hg)	78.96 ± 6.22	74.2 ± 9.45	0.060 (NS)
SDRR (milliseconds)	71.27 ± 27.40	21.93 ± 3.53	0.000^⁕^
LF/HF ratio	0.98 ± 1.07	1.43 ± 0.40	0.023^⁕^

Table 3 shows the correlation of HRV parameters (SDRR and the LF/HF ratio) with cardiovascular parameters (pulse and mean blood pressure). There was a positive correlation between SDRR and the LF/HF ratio with pulse and a negative correlation with mean blood pressure.

**Table 3:** Correlation of SDRR and LF/HF with pulse and mean blood pressure NS=non-significant, SDRR=standard deviation of the RR interval, LF/HF=ratio of low frequency to high frequency

	SDRR (milliseconds)		LF/HF Ratio	
N=25	Correlation Coefficient	p-value	Correlation Coefficient	p-value
PULSE (bpm)	0.229	0.271 (NS)	0.031	0.884 (NS)
MEAN BLOOD PRESSURE (mm of hg)	-0.065	0.758 (NS)	-0.246	0.236 (NS)

## Discussion

The present findings revealed that there was a significant difference in the mean pulse rate between cases and controls. The cases showed a significantly higher pulse rate than the control. The mean ± SD SDRR (milliseconds) was significantly lower among the cases as compared to the controls. This hints at sympathetic preponderance among the cases of syncope. The findings correlated well with those of some previous researchers [8-11]. This parameter was related to the vagal influence of the heart, which shows that the vagal tone of the heart is affected in subjects who have vasovagal syncope. These results also hint at the pathophysiological mechanism of fainting and provide means of evaluating patients with syncope. These act as physiological biomarkers to explore the influence of the autonomic nervous system on sinus node function, particularly the vagal control of the heart [11-13]. However, the findings of a few researchers were contradictory to the present findings [14]. There was no statistically significant difference in the mean value of systolic and diastolic blood pressure between the cases and the controls.

The LF/HF ratio showed significantly higher values among the cases as compared to the controls. This showed a sympathetic predominance among the cases of syncope. These findings correlated well with those of previous researchers [11,15]. The sympathetic overactivity may be due to compensatory autonomic modulations arising due to the hemodynamic alterations [16]. This finding suggests that syncope is characterized by an increase in noradrenergic tone and sympathetic response to a prolonged upright position, with compensatory cardiac sympathetic overactivity. The arterial baroreceptors located in the carotid sinus and aortic arch during a syncopal attack transmit signals to the nervous system and result in reflex-increased sympathetic output and parasympathetic inhibition [17].

There was a positive correlation between the mean pulse rate and SDRR (r=0.229) as well as the mean pulse rate and LF/HF ratio (0.031) among the cases. However, this was not found to be statistically significant. It was found that there was a negative correlation between mean blood pressure and SDRR (r=-0.065) and mean blood pressure and the LF/HF ratio (-0.246) among the cases. This too was not significant statistically.

The limitation of our study was the less sample size due to the unavailability of subjects with unexplained syncope within a limited period of time. This project was approved by the Indian Council of Medical Research (ICMR)-Short Term Studentship Program, 2022, which had to be completed within a short period of time.

## Conclusions

The cases of unexplained syncope showed significantly decreased mean SDRR values and an increased LF/HF ratio, suggesting sympathetic predominance. This could be attributed to compensatory hemodynamic changes. These findings can be used as potential physiological biomarkers to diagnose patients with unexplained syncope and may help in their prognosis, which needs to be reaffirmed by studies with a robust study design and a larger sample size.
